# Quetiapine versus aripiprazole in children and adolescents with psychosis - protocol for the randomised, blinded clinical Tolerability and Efficacy of Antipsychotics (TEA) trial

**DOI:** 10.1186/1471-244X-14-199

**Published:** 2014-07-11

**Authors:** Anne Katrine Pagsberg, Pia Jeppesen, Dea Gowers Klauber, Karsten Gjessing Jensen, Ditte Rudå, Marie Stentebjerg-Olesen, Peter Jantzen, Simone Rasmussen, Eva Ann-Sofie Saldeen, Maj-Britt Glenn Lauritsen, Niels Bilenberg, Anne Dorte Stenstrøm, Jesper Pedersen, Louise Nyvang, Sarah Madsen, Marlene B Lauritsen, Ditte Lammers Vernal, Per Hove Thomsen, Jakob Paludan, Thomas M Werge, Kristian Winge, Klaus Juul, Christian Gluud, Maria Skoog, Jørn Wetterslev, Jens Richardt M Jepsen, Christoph U Correll, Anders Fink-Jensen, Birgitte Fagerlund

**Affiliations:** 1 Child and Adolescent Mental Health Center, Mental Health Services - Capital Region of Denmark & Faculty of Health Science University of Copenhagen, Nordre Ringvej 69, Glostrup DK- 2600, Denmark; 2 Child and Adolescent Psychiatry Research Unit, Region of Southern Denmark, University of Southern Denmark, Sdr. Boulevard 29, Odense C DK-5000, Denmark; 3 Mental Health Centre for Child and Adolescent Psychiatry Region Zealand, Research Unit, University of Copenhagen, Smedegade 16, Roskilde DK-4000, Denmark; 4 Mental Health Centre for Child and Adolescent Psychiatry North Denmark Region, Research Unit, University of Aalborg, Mølleparkvej 10, Aalborg DK-9000, Denmark; 5 Mental Health Centre for Child and Adolescent Psychiatry Central Denmark Region, Research Unit, University of Aarhus, Harald Selmers Vej 66, Risskov DK- 8240, Denmark; 6 Psychiatric Centre Sct. Hans, Research Institute for Biological Psychiatry, University of Copenhagen, Boserupvej 2, Roskilde DK- 4000, Denmark; 7 Department of Neurology, Bispebjerg Movement Disorders Biobank, Bispebjerg University Hospital, Bispebjerg Bakke 23, DK-2400 Copenhagen, NV, Denmark; 8 Department of Pediatrics, Rigshospitalet University Hospital, Blegdamsvej 9, DK- 2100 Copenhagen Ø, Denmark; 9 Copenhagen Trial Unit, Centre for Clinical Intervention Research, Rigshospitalet, Copenhagen University Hospital, Blegdamsvej 9, DK 2100 Copenhagen, Denmark; 10 Centre for Neuropsychiatric Schizophrenia Research and Lundbeck Foundation Centre for Clinical Intervention and Neuropsychiatric Schizophrenia Research, Psychiatric Centre Glostrup, Ndr. Ringvej 29-67, DK- 2600 Glostrup, Denmark; 11 Hofstra North Shore Long Island Jewish School of Medicine and The Zucker Hillside Hospital, New York, 75-59 263rd Street, Glen Oaks, 11004 New York, USA; 12 Laboratory of Neuropsychiatry, Department of Neuroscience and Pharmacology, University of Copenhagen and Psychiatric Centre Copenhagen, University Hospital Copenhagen, Edel Sauntes Allé 10, DK-2100 København Ø, Denmark

**Keywords:** Antipsychotics, Quetiapine, Aripiprazole, Psychosis, Schizophrenia, Children, Adolescents, Randomised trial, Benefits, Harms

## Abstract

**Background:**

The evidence for choices between antipsychotics for children and adolescents with schizophrenia and other psychotic disorders is limited. The main objective of the Tolerability and Efficacy of Antipsychotics (TEA) trial is to compare the benefits and harms of quetiapine versus aripiprazole in children and adolescents with psychosis in order to inform rational, effective and safe treatment selections.

**Methods/Design:**

The TEA trial is a Danish investigator-initiated, independently funded, multi-centre, randomised, blinded clinical trial. Based on sample size estimation, 112 patients aged 12-17 years with psychosis, antipsychotic-naïve or treated for a limited period are, 1:1 randomised to a 12- week, double-blind intervention with quetiapine versus aripiprazole. Effects on psychopathology, cognition, health-related quality of life, and adverse events are assessed 2, 4, and 12 weeks after randomisation. The primary outcome is change in the positive symptom score of the Positive and Negative Syndrome Scale. The recruitment period is 2010-2014.

**Discussion:**

Antipsychotics are currently the only available pharmacologic treatments for psychotic disorders. However, information about head-to-head differences in efficacy and tolerability of antipsychotics are scarce in children and adolescents. The TEA trial aims at expanding the evidence base for the use of antipsychotics in early onset psychosis in order to inform more rational treatment decisions in this vulnerable population. Here, we account for the trial design, address methodological challenges, and discuss the estimation of sample size.

**Trial registration:**

ClinicalTrials.gov: NCT01119014

## Background

Early onset psychosis (EOP, onset before age 18 years) is considered grave and may have worse outcomes compared to adult onset psychosis [1,2]. In adults, antipsychotics have proven more effective than placebo in improving psychotic symptoms and preventing relapse [3,4] and comparisons of benefits and harms of different antipsychotic drugs have been assessed in large meta-analyses [5,6]. In children and adolescents with EOP, placebo-controlled trials and, especially, active-controlled trials are limited [7-10].

Seven primarily industry-sponsored randomised placebo-controlled trials covering a total of 1198 children and adolescents with schizophrenia have been published: six trials investigated effects of the second-generation antipsychotics (SGAs) aripiprazole [11], olanzapine [12], risperidone [13], paliperidone [14], quetiapine [15], and ziprasidone [16] in adolescents with schizophrenia. One trial compared the first-generation antipsychotic (FGA) haloperidol with placebo in children with schizophrenia [17]. Six of these seven trials showed superiority of the antipsychotic compared with placebo. The ziprasidone trial was terminated due to lack of efficacy. Only 12 randomised clinical trials (RCTs) compared different antipsychotics in the treatment of EOP. Five RCTs were open-label head-to-head studies investigating olanzapine versus (vs) risperidone (n = 44, including adolescents and young adults aged 16-28 years) [18]; risperidone vs olanzapine vs quetiapine (n = 30) [19]; olanzapine vs quetiapine (n = 50) [20]; quetiapine vs risperidone (n = 22) [21]; and olanzapine vs risperidone (n = 25) [22]. Six RCTs were double-blind, head-to-head trials investigating clozapine vs haloperidol (n = 21) [23]; clozapine vs olanzapine (n = 39) [24]; clozapine vs olanzapine (n = 25) [25]; risperidone vs olanzapine vs haloperidol (n = 50, including both non-affective and affective psychotic disorders) [26]; olanzapine vs risperidone vs molindone (n = 119) [27]; and a double-blind RCT (n = 126) comparing paliperidone vs aripiprazole [28]. One older RCT assessed thiothixene vs thioridazine (n = 21) [29]. Across these trials, only clozapine has shown superiority compared with other antipsychotics (haloperidol or olanzapine) when used for treatment-resistant early onset schizophrenia [23-25]. Each of these head-to-head RCTs included small to medium size patient samples (total n = 572). In comparison, a recent meta-analysis of antipsychotic drugs in adult schizophrenia included data from 43,049 patients [6]. It is problematic that the use of antipsychotics in EOP to a large extent is based on extrapolations from trials conducted in adults, considering that the prevalence of adverse reactions and treatment resistance may be higher in children and adolescents [20,30-32].

Outcome of antipsychotic treatment in psychosis is typically assessed with validated psychopathology scales and unsolicited adverse effect reporting, supplemented with usually few validated adverse events scales and some physical health parameters. However, other potential outcomes are also relevant for the prognosis of EOP, including cognitive deficits [33-41] and health-related quality of life (HRQoL) [42]. It has been difficult to observe consistent effects of antipsychotics on these outcomes in adults [38,39,43-45], and in the few studies conducted in EOP [46-50].

The Food and Drug Administration (FDA) has approved the use of quetiapine and aripiprazole in the treatment of schizophrenia from age 13 years, but no trials have compared the two drugs in EOP [51]. Here, we present the trial protocol of an investigator-initiated and independently funded RCT comparing the effects of quetiapine vs aripiprazole in EOP.

### Objectives

The primary objective of the TEA (Tolerability and Efficacy of Antipsychotics) trial is to compare quetiapine vs aripiprazole in the treatment of EOP with regards to efficacy (psychopathology, cognition, HRQoL) and tolerability (motor adverse events (AEs); metabolic AEs; hormonal AEs; cardiac AEs, suicidal ideation; and other AEs) in a randomised, blinded design.

### Hypotheses

The TEA trial tests the null hypothesis that there are no significant differences between quetiapine vs aripiprazole in EOP after 12 weeks of treatment on the primary outcome, Positive and Negative Syndrome Scale (PANSS) positive symptoms (PANSS-P) [52]. Due to different receptor profiles and prior data [7,8,11,15] we expect specific differences in secondary outcomes primarily reflected in adverse effect assessments with aripiprazole causing more akathisia than quetiapine; and quetiapine causing more sedation, weight gain, and metabolic adverse effects than aripiprazole.

## Methods/Design

### RCT design

The TEA trial is a Danish investigator-initiated, independently funded, randomised, blinded, multi-centre superiority trial. Patients (n = 112) are randomised to a 12 weeks intervention period with quetiapine vs aripiprazole.

### Participants

Patients are recruited from seven child- and adolescent mental health centres covering all Danish university clinics nationwide (University Hospitals of Copenhagen (4 centres), Southern Denmark (1 centre), Aarhus (1 centre), and Aalborg (1 centre)).

#### ***Patient inclusion criteria***

Inclusion criteria: 1) Children and adolescents aged 12-17 years (both inclusive), both sexes; 2) in- or outpatients; 3) meeting the criteria for ICD-10 [53] psychosis diagnoses (non-organic, non-drug-induced): F20 (Schizophrenia), F22 (Persistent delusional disorders), F23 (Acute and transient psychotic disorders), F24 (Induced delusional disorders), F25 (Schizoaffective disorders), F28/F29 (Other/Unspecified nonorganic psychosis), F30.2 (Mania with psychotic symptoms), F31.2 (Bipolar affective disorder, current episode manic with psychotic symptoms), F31.5 (Bipolar affective disorder, current episode severe depression with psychotic symptoms), F32.3 (Severe depressive episode with psychotic symptoms), or F33.3 (Recurrent depressive episode, current episode severe with psychotic symptoms). The diagnosis is verified by Schedule for Affective Disorders and Schizophrenia for School-Age Children Present and Lifetime version (K-SADS-PL) 4 weeks after inclusion into the trial [54]; 4) clinical indication for antipsychotic treatment; 5) presence of psychotic symptoms scoring ≥4 on at least one of the following PANSS items: P1 (delusions), P2 (conceptual disorganisation), P3 (hallucinations), P5 (grandiosity), P6 (suspiciousness/persecution), or G9 (unusual thought content); as well as a total PANSS score >60 points; 6) antipsychotic-naïve or limited exposure (i.e., no more than 12 months in the past year for psychosis, or no more than 1 week lifetime for any non-psychotic indication); and 7) written informed consent by caretakers.

#### ***Patient exclusion criteria***

Exclusion criteria: 1) Compulsory treatment; 2) drug-induced or organic psychosis; 3) severe chronic somatic illness or a history of severe head trauma; 4) pregnancy or lactation; 5) substance dependence (ICD-10 F1X.2 dependence syndrome) within the last year; 6) allergy towards the investigational drugs or lactose intolerance; 7) lack of informed consent.

### Randomisation and blinding

Randomisation of patients to treatment is carried out centrally by the Copenhagen Trial Unit at the Capital Region Pharmacy using a computer-generated allocation sequence with a block size unknown to the investigators. To optimise the comparability between treatment groups, randomisation is stratified by two factors: PANSS-P score (i.e., ≤20 points or >20 points), and age (i.e., 12-14 years or 15-17 years). Computer-generated and sealed allocation sequence lists are prepared according to the stratification variables and the trial medication packages are distributed accordingly. The interventions are blinded to participants, caregivers, outcome assessors, statisticians, conclusion drawers, as well as all investigators and staff involved in the trial, except for the pharmacy personnel who pack and distribute the two study drugs and the data managers at the Copenhagen Trial Unit.

### Interventions and assessments

During the first 9 days, the assigned antipsychotic is uptitrated in fixed dose steps, with slower titrations and dose adjustments if clinically indicated (see below). The target doses are quetiapine 600 mg/day vs aripiprazole 20 mg/day. However, if needed, dosing is flexible (quetiapine: 50-800 mg/day; aripiprazole 2.5-30 mg/day). Beneficial and harmful effects are assessed at three time points during the intervention period, i.e., weeks 2, 4, and 12.

### Post-randomisation exclusions

Patients are excluded if there is a significant worsening of their clinical state during the course of the trial (i.e., increases of 20% or more from baseline on the PANSS total score).

### Outcomes

The primary outcome is positive symptoms measured on the PANSS-P scale. Secondary outcomes are 1) other psychopathology measures assessed on PANSS negative and general scales; Dimensions of Psychosis Instrument (DIPI) [55]; Clinical Global Impressions - Severity/Improvement/Efficacy (CGI-S/I/E) [56]; Global Assessment of Psychosocial Disability (GAPD) [53]); 2) cognition and cognitive daily functioning measured on Brief Assessment of Cognition in Schizophrenia (BACS) Global Score [57]; Schizophrenia Cognition Rating Scale, Danish version (SCoRS-DK) [58]; Behavioural Rating Inventory of Executive Functions (BRIEF) [59]); 3) HRQoL measured on KIDSCREEN-52 [60]; 4) adverse reactions measured on ‘Udvalget for Kliniske Undersøgelser’ Side Effect Rating Scale (UKU) [61]; Abnormal Involuntary Movement Scale (AIMS) [62]; Simpson Angus Scale (SAS) [63]; Barnes Akathisia Rating Scale (BARS) [64]; cardiac adverse reactions (such as QT-interval prolongation on Electrocardiography (ECG)); somatic events (blood pressure, pulse, body weight, height, body mass index, abdominal circumference, abnormal laboratory test results (fasting blood glucose, insulin and lipid levels, prolactin, other general blood tests)); suicidal ideation measured by K-SADS-PL (specific items); adherence (daily registration by caretakers or staff and antipsychotic blood levels); substance use (interview and urine testing) (Table 1).

**Table 1:** TEA RCT, schedule for outcome assessments during 12 weeks of blinded intervention

**Assessments**	**Inclusion (week 0)**	**Follow-up (week 2)**	**Follow-up (week 4)**	**Follow-up (week 12)**
**Psychopathology and adverse reactions rating scales** PANSS, DIPI, CGI-S, CGI-I, CGI-E, GAPD, UKU, AIMS, SAS, and BARS	X	X	X	X
**Suicidal ideation** K-SADS-PL specific items	X	X	X	X
**Cognition, cognitive daily and executive function** BACS, SCoRS-DK and BRIEF	X			X
**Somatic examination** Standard clinical examination, blood pressure, pulse, weight, height, BMI, and abdominal circumference.	X	X	X	X
**Health-related quality of life** Questionnaire: KIDSCREEN-52	X			X
**Laboratory tests** (see list below)	X		X	X

AIMS = Abnormal Involuntary Movement Scale; BACS = Brief Assessment of Cognition in Schizophrenia; BARS = Barnes Akathisia Rating Scale; BMI = Body Mass Index; BRIEF = Behavioural Rating Inventory of Executive Functions; CGI = Clinical Global Impression (-I = Improvement -S = Severity -E = Efficacy); DIPI = Dimensions of Psychosis Instrument; GAPD = Global Assessment of Psychosocial Disability; HRQoL = Health related quality of life; K-SADS-PL = Kiddie-SADS-Present and Lifetime Version; KIDSCREEN-52 = Health related quality of life questionnaire for children and young people and their parents; PANSS = Positive and Negative Syndrome Scale; SCoRS-DK = Schizophrenia Cognition Rating Scale – Danish version; UKU = ‘Udvalget for Kliniske Undersøgelser’ Side Effect Rating Scale.

Laboratory tests: Blood tests (fasting): triglycerides; total cholesterol; high-density and low-density lipoproteins; glucose; insulin; prolactin; creatinphosphokinase; haemoglobin; leukocyte cell and differential count; thrombocyte cell count; sodium; potassium; creatinine; aspartate amino transferase; alkaline phosphatases; thyroid stimulating hormone; vitamin D; omega 3 fatty acids; genetic material (DNA). Blood samples to analyse serum values of antipsychotics are drawn at week 4 and 12, and saved for later analysis. Urine tests: pregnancy test (at baseline and at follow-up if suspicion of pregnancy), screening for medication and substance abuse (at baseline and at follow-up if suspicion of substance use). ECG: Standard leads.

### Antipsychotic drugs

#### ***Quetiapine and aripiprazole***

Quetiapine is a low-affinity dopamine D2 receptor antagonist [65]. Aripiprazole has a high affinity for dopamine D2 receptors but with a partial agonist mode of action at this receptor [66]. Both compounds are approved by FDA and the European Medicines Agency (EMA) for the treatment of schizophrenia in adults. Aripiprazole is approved by the FDA for use in schizophrenia for ages 13-17 years, and in Denmark and the rest of the EU it has obtained authorisation for use in adolescent schizophrenia for ages of 15-17 years by the EMA [67]. Quetiapine has FDA approval for the treatment of schizophrenia for ages 13-17 years, but it has not been approved by EMA for use below age 18 years. In the present trial, quetiapine and aripiprazole were selected since these compounds are frequently used in clinical child and adolescent psychiatric practice, and because it remains unclear whether their different receptor binding profiles can be related to differences in clinical outcomes in EOP. In adults, a recent Cochrane systematic review found no significant differences between quetiapine vs aripiprazole (global state (n = 991, 12 RCTs); PANSS-P (n = 583, 7 RCTs); leaving the study early for any reason (n = 168, 2 RCTs), or general extra pyramidal symptoms (n = 348, 4 RCTs); all low to very low quality evidence). Results were significantly in favor of aripiprazole regarding Quality of Life (n = 100, 1 RCT, mean difference 2.60, 95% CI 1.31 to 3.89), however, evidence was rated very low quality [51]. Notably, all studies were conducted in China and in adult Chinese patients. Currently, no single Western RCT has compared quetiapine with aripiprazole either in adults or in children and adolescents.

#### ***Quetiapine and quetiapine extended release***

Studies have shown that quetiapine 200-800 mg/day improves psychotic symptoms in patients with EOP (age 11-17 years) [68], and 300-800 mg/day showed acceptable long-term (88 weeks) safety and tolerability (n = 10, age 12-16 years [69]). Patients aged 10–17 years (n = 27) tolerated quetiapine doses of 400 mg twice daily, with no serious AEs and no unexpected events reported, and compared to a parallel adult population there were similar pharmacokinetic, safety, and tolerability profiles by dose escalation, suggesting that no dosage adjustment is required when treating patients of these ages [70]. One 6-week, double-blind, placebo-controlled RCT of quetiapine 400 or 800 mg/day in 220 adolescents with schizophrenia aged 13-17 years found significant improvements in PANSS total score changes at both quetiapine doses compared with placebo, and quetiapine was generally well tolerated with a profile broadly similar to that reported in adult populations [15]. A 12-week open label study of quetiapine in 56 adolescents with schizophrenia spectrum disorder found a significant reduction of PANSS total and particularly positive symptom scores [71]. It is generally assumed that younger patients require lower doses of antipsychotics than adults. However, for quetiapine this may be true for younger children with lower body weight, but adolescents typically require rapid titration to the same, or even higher, dose levels than adults for optimal clinical response [32]. Higher doses of quetiapine than those recommended by the compound producer have proven to be effective and safe in adolescents [26]. Extended release quetiapine allows a once-daily dosing and has been proven effective and well tolerated in adults with schizophrenia in a randomised, placebo-controlled trial [72]. This formulation will be used in the present trial in order to match the once a day dosing of aripiprazole.

We use a 9-day fixed titration phase with a final quetiapine extended release dose of 600 mg/day (Table 2). If needed for efficacy, the possible maximum dose can be titrated up to 800 mg/day, or titrated down to a lower dose level if not tolerated (minimum 50 mg/day).

**Table 2:** Schedule of dosing regimen

**Level**	**1**		**2**		**3**		**4**		5*	6**
**Day**	**1**	**2**	**3**	**4**	**5**	**6**	**7**	**8**	**9**	**…**
**Quetiapine ER (mg)**	50	50	100	100	200	200	400	400	600	800
**Aripiprazole (mg)**	2.5	2.5	5	5	10	10	15	15	20	30
**Number of capsules**	1	1	2	2	1	1	2	2	3	4
**Quetiapine ER:**	**●**	**●**	**●●**	**●●**	**●**	**●**	**●●**	**●●**	**●●●**	**●●●●**
**Aripiprazole:**	**●**	**●**	**●○**	**●○**	**●**	**●**	**●○**	**●○**	**●●○**	**●●○○**

Due to unparalleled strengths in formulations of quetiapine ER and aripiprazole, the use of capsules filled with a neutral ingredient (lactose) is needed in order to have the same number of capsules for each compound at each level. ER=extended release.

*Level 5 = final level; **Level 6 = maximal level (if needed);

**●**Capsules with active compound; **○** Capsules without active compound.

#### ***Aripiprazole***

The safety of aripiprazole used in children and adolescents from the age of 7 years has mainly been documented in open-label or naturalistic studies of disorders other than psychosis, i.e., Tourette’s syndrome or developmental disorders, using doses in the mean range of 5-17 mg/day [73-78]. A double-blinded multi-centre RCT compared aripiprazole 10 mg/day and 30 mg/day with placebo in 302 adolescents (aged 13-17 years) with schizophrenia [11]. The trial authors concluded that 6 weeks treatment with aripiprazole was generally well tolerated and lead to significant improvements in psychotic symptoms compared with placebo. An open-label trial with 21 patients aged 10-17 with bipolar or schizophrenia spectrum disorders compared daily doses of 20, 25 or 30 mg aripiprazole in a 12-days titration phase, followed by a 14-days fixed dose phase. Effectiveness and criteria for tolerability were met for all doses, and no adverse reactions met the regulatory criteria for serious adverse reactions [79].

We use a 9-day fixed titration phase with a final aripiprazole dose of 20 mg/day (Table 2). If needed for efficacy, the possible maximum dose can be titrated up to 30 mg/day. If final doses are not tolerated, patients can be titrated down to a lower dose level (minimum 2.5 mg/day).

#### ***Concomitant medications***

Patients will not be allowed any concomitant antipsychotic medication before all trial assessments at week 12 have been conducted. Concomitant medication of any kind beyond trial medications will be recorded throughout the trial intervention period.

### Sample size estimation

The sample size estimation is based on the primary outcome measure, the PANSS-P score. In 2009, when the TEA trial was designed, the estimated standard deviation (SD) for PANSS-P was based on the few studies that up until then had investigated efficacy of the trial drugs (quetiapine or aripiprazole) in patients below 18 years of age with psychosis [11,20,71]. The three studies reported mean PANSS-P in patients treated with quetiapine or aripiprazole with SDs ranging from 4.1 to 7.4, or standard error (SE) for change of PANSS-P from entry to end of treatment of around 0.6 (corresponding to SD = 5.9). Furthermore, in our previous case-control study of patients comparable to the TEA participants, consisting of Danish minimally medicated children and adolescents with psychosis aged 12-18 years, the SD of PANSS-P at the time of first-episode psychosis was within the above range, SD = 5.1 [80,81]. Consequently, we used a conservative estimate of the (at that time) highest reported SD of 7.4 in the original sample size estimation. Since then, however, more studies on aripiprazole and quetiapine in early onset psychosis point toward lower SDs on the PANSS-P ranging from 4.3 to 6.3 [15,21,28] (see all relevant studies in Table 3). A weighted mean SD of PANSS-P based on data from all 6 studies in Table 3 equals 5.48 for all SDs, and 5.68 for SDs of change scores. Hence, based on the entirety of the available data, we updated our sample size calculation based on a SD of 5.7 on the PANSS-P scale.

**Table 3:** Data from trials on quetiapine or aripiprazole in patients below 19 years with psychosis used for final power calculation

**Study (reference)**	**Design and population**	**Intervention**	**ITT population N**	**PANSS-P scores baseline (SD)**	**PANSS-P scores follow-up (SD)**	**Change PANSS-P scores from baseline to follow-up (SD)**
Schimmelman et al. 2007 [71]	Open Label	Quetiapine	N=52	Quetiapine:	Quetiapine:	Quetiapine:
	Non-RCT					
	12 weeks	200-800 mg		21.4 (5.2)	14.5 (7.4)	-6.9 (na)
	Schiz. spectrum					
	Most drug-naïve					
	12-17 years					
Findling et al. 2008 [11]	Double-blind RCT 6 weeks	Aripiprazole 10 mg/30 mg vs placebo	N=302 (100 vs 100 vs 102)	Aripiprazole 10 mg: 22.1 (SD=5.0)	Aripiprazole 10 mg: 14.5 (SE=0.6 i.e. SD=6.0*)	Aripiprazole 10 mg: -7.6 (SE=0.6 i.e. SD=6.0*)
	Schiz. 13-17 years					
				Aripiprazole 20 mg: 23.5 (SD=5.0)	Aripiprazole 20 mg: 15.4 (SE=0.6 i.e. SD=5.9*)	Aripiprazole 20 mg: -8.1 (SE=0.6 i.e. SD=5.9*)
Arango et al. 2009 [20]	Open label RCT	Quetiapine vs Olanzapine	N=50	Quetiapine:	Quetiapine:	Quetiapine:
	6 months		(24 vs 26)	23.3 (7.3)	15.1 (4.1)	-8.2 (na)
	Psychotic disorders					
	Most drug-naïve	flexible doses				
	12-18 years					
Swadi et al. 2010 [21]	Open label RCT	Quetiapine up to 800 mg vs risperidone up to 6 mg	N=22	na	na	Quetiapine:
	6 weeks		(11 vs 11)			-9.6 (5.5)
	First episode					
	psychosis					
	Below 19 years					
Findling et al. 2012 [15]	Double-blind RCT	Quetiapine 400 mg/800 mg vs placebo	N=220 (73 vs 74 vs 73)	Quetiapine 400 mg: 23.3 (5.8) Quetiapine 800 mg: 23.8 (4.8)	na	Quetiapine 400 mg:
	6 weeks Schiz.					-8.6 (SE 0.7, i.e. SD=6.3*)
	13-17 years					Quetiapine 800 mg:
						-9.3 (SE 0.6, i.e. SD=5.1*)
NCT01009047 2013 [28]	Double-blind RCT	Paliperidone ER 3-9 mg vs Aripiprazole 5-15 mg	N=226 (112 vs 114)	Aripiprazole: 22.5 (4.3)	na	Aripiprazole week 8:
	8 weeks/26 weeks					-6.2 (4.9)
	Schiz.					Aripiprazole week 26:
	12-17 years					-8.3 (6.1)

Data on PANSS positive scores and standard deviations from these studies were considered in the sample size estimation of the TEA study. Weighted average of all SDs=5.48 and of change in SD=5.68. ITT=Intention-to-treat. PANNS-P=Positive and Negative Syndrome Scale – positive score. SD=standard deviation. SE=standard error. na=not available. ER=extended release formulation. vs=versus. Schiz.=Schizophrenia. *SD=SE√n.

Based on results from a 24 weeks RCT comparing olanzapine with quetiapine that found a mean difference in PANNS-P scores of 4 points in favour of olanzapine (a difference that did not reach significance probably due to the small group size of 25 patients each) [20], we decided that a minimal clinically relevant difference between outcome responses of quetiapine vs aripiprazole after (the shorter period of) 12 weeks of treatment would be 3 points on the PANSS-P. Hence, using a power of 80% and a two-sided alpha of 5%, and expecting a SD of 5.7 on the PANSS-P, the required sample size necessary to detect or reject a difference of at least 3 points on PANSS-P was estimated as follows (N = total sample size, SD of the primary outcome measure = 5.7 (we assume SD_1_ = SD_2_), α = type 1 error = 0.05, β = type 2 error = 0.20, MIREDIF = minimal relevant difference of outcome measure = 3, z = fractiles in normal distribution): N = 2 * (SD_*1*_^*2*^ *+ SD*_*2*_^*2*^) * (z_α*/2*_ *+ z*_β_*)/MIREDIF*^*2*^ *= 112.* Accordingly, we should strive to include a total of 112 patients, equivalent to about 56 patients in each of the intervention groups.

### Data analysis

Data will be blinded during the analysis, and the blinding will not be broken before two conclusions are drawn (i.e., the first assumes treatment A to be quetiapine and treatment B to be the aripiprazole; the second is based on the reverse assumption, i.e., that treatment A is aripiprazole and B is quetiapine) [82]. A detailed protocol for the statistical analysis plan will be presented in a separate paper. In short, the primary analysis will be according to the modified intention-to-treat principle [83] with adjustment for the protocol specified stratification variables [84]. Data missingness will be investigated and if deemed necessary, generally when missingness is not completely at random, a suitable multiple imputation method will be used [85-92]. The data will be analysed with a two-sided statistical testing and p-values will be assessed at a significance level of 5% with correction for multiple testing according to Holm [93]. Covariates considered relevant to adjust for in comparative analysis will be incorporated in the statistical model. We aim at analysing the data as trajectories including multiple assessments whenever possible.

### Ethical considerations

As the trial is investigating medicinal products and the participants are under 18 years of age, a surrogate written informed consent is required from the holders of custody. The trial is registered at ClinicalTrials.gov: NCT01119014, EudraCT: 2009-016715-38, and approved by Danish Medicines Agency: 2612-4168, The Ethics Committee of Capital Region: H-3-2009-123 and Danish Data Protection Agency: 2009-41-3991. All procedures for registering and reporting adverse reactions and adverse events will follow the regulations and guidelines from the Danish Health and Medicines Authority and the Ethics Committee.

## Discussion

### Legitimacy of the trial

The use of antipsychotics in children and adolescents is rising in many countries [94-97]. In Denmark, the number of persons aged 0-18 years treated with antipsychotics (all indications) increased by a factor four from 1999 to 2012 [98]. Secondly, off-label use is prevalent. In the United States, five newer antipsychotics (aripiprazole, olanzapine, paliperidone, quetiapine, and risperidone) are currently approved for schizophrenia in children aged 13 to 17, but in the EU until recently only one compound (aripiprazole) has been approved for the use in schizophrenia from age 15 (in June 2014 paliperidone was approved for the same indication [99]). The off-label use in paediatric populations is of major concern to the EMA which calls for randomised trials [100]. Thirdly, in child- and adolescent psychiatry only seven double-blinded RCTs comparing antipsychotics for the treatment of child- and adolescent psychosis have been carried out, and since the clinical effects vary between compounds and among patients, there is no doubt that RCTs including large samples are lacking in this field. Specifically, to date no RCTs investigated quetiapine vs aripiprazole in EOP. Notably, even in adults, no RCT outside of China has compared quetiapine vs aripiprazole for the treatment of psychosis either [51]. Moreover, more frequent and severe adverse effects that can have potential long-term consequences, i.e., metabolic syndrome, may be expected in young patients [8,101].

### Time frame

A challenge in clinical practice and in trials is to identify the relevant time frame for assessing the effectiveness of antipsychotic treatment. We decided to investigate as our primary outcome the antipsychotic response after 12 weeks of treatment. In clinical practice, the usual routine is to continue treatment with an antipsychotic drug for a period of at least 4 - 6 weeks to evaluate the antipsychotic response. This individual drug trial period is partly arbitrarily defined [102-105] based on older preclinical studies [106]. However, brain imaging studies of both FGAs and SGAs have shown that dopamine D2 receptor occupancy occurs within hours after administration [107,108], and clinical antipsychotic effects can be distinguished from non-specific sedative effects within the first 24 hours of treatment [104]. Studies in adults have demonstrated that the largest effect of antipsychotics occur during the first four weeks of treatment [103,109], and that the early response after 4 weeks can predict long-term effects [105]. The only two studies on this issue in EOP found that early response to aripiprazole after 3 weeks predicted well ultimate response at 6 weeks in an RCT including youth with schizophrenia [51,110,111] and that early response to mixed SGAs after 4 weeks predicted response at week 12 in naturalistically treated youth with schizophrenia spectrum disorders [112]. In order to further investigate the early clinical response as a predictor of later clinically significant effects on psychopathology, we decided to measure treatment responses after 2, 4 and 12 weeks of treatment in the randomised, blinded design, and to measure the long-term effects in a naturalistic design with follow-up at 1 year after randomisation.

### Sample size estimation

The key consideration in designing a RCT is to know the size of a sample needed to obtain limited probability of type 1 (false positive) and type 2 (false negative) errors. The sample size estimation is based on choices regarding an acceptable risk of type 1 and 2 errors, on assumptions regarding the variation in outcome measures, and finally on the estimation of the minimal relevant difference in clinical effect between the interventions under the trial. Thus, the following four variables were considered:

1) α or type 1 error (the probability of rejecting the null hypothesis when it is actually true) is usually set at 0.05 two-sided. If we find a difference below this level we may declare a significant difference, knowing that there will still be a 5% risk that the null hypothesis of no difference is correct.

2) We decided in our updated sample size estimation to use a value of 80% for power, which is standard and translates into accepting a 20% risk that we will miss a true difference of the anticipated magnitude between the two compounds of (in this case) 3 points on the PANSS-P scale.

3) The expected variation in the primary outcome measure PANSS-P was initially driven by the only three relevant RCTs (upper three studies in Table 3) that were available in 2009 [11,20,71]. During our recruitment in the TEA trial, more informative studies have appeared (lower three studies in Table 3), i.e., two published RCTs [15,21] and one RCT reported to clinicaltrials.gov [28]. Based on a weighted mean SD of change in PANSS-P scores from all six trials, we used in our updated sample size calculation a SD of 5.7 on the PANSS-P scale.

4) MIREDIF should represent a meaningful minimal difference in response in the sense that it would justify a choice between two interventions in a clinical setting. The PANSS-P scale scores range from 7 to 49, and the few relevant RCTs already conducted found that the mean PANSS-P score after 6 weeks to 6 months of treatment is around 15 in the aripiprazole or quetiapine groups with a mean PANSS-P score change in the range 6.2–9.6. For the TEA trial, we decided in our updated sample size estimation, based on data from a head-to-head trial comparing olanzapine vs quetiapine in EOS [20], that it would be clinically relevant to be able to detect a difference of 3 points in outcome score on the PANSS-P scale between the two drugs.

Underpowered trials bear the risk of both type 1 and type 2 errors. On the other hand, an unnecessarily large sample size wastes resources, from participants and researchers and may increase risk of type 1 error, by rejecting the null hypothesis in favor of small significant effects that are clinically meaningless [113]. Challenges regarding sample size calculations often, as in this trial, stem from uncertainty concerning parameter estimates due to limited information prior to the launch of the trial. Deciding on the values of the parameters included in the sample size estimation are crucial, since relatively small changes in best estimates of variance of the outcomes, and in choice of size of clinical relevant differences in outcomes, power or α demands can alter the sample size needed. Table 4 gives some examples, including both our original and our updated sample size estimation, and shows the effects of variation in variables on the sample size.

**Table 4:** Sample size estimation

	**β**	**SD**	**MIREDIF**	**Effect size**	Calculations: N = 4 * (z_**α/2**_ **+ z**_**β**_**)**^**2**^ * (SD_**1**_**/MIREDIF)**^**2 #**^	**N**
*TEA original*	*0.10*	*7.4*	*3.5*	*0.47*	N = 42 * (7.4 / 3.5)^*2*^	*188*
TEA smaller MIREDIF	0.10	7.4	2.0	0.27	N = 42 * (7.4 / 2.0)^2^	575
TEA larger MIREDIF	0.10	7.4	5.0	0.67	N = 42 * (7.4 / 5.0)^2^	92
TEA larger SD	0.10	10.0	3.5	0.35	N = 42 * (10.0 / 3.5)^2^	343
TEA smaller SD	0.10	5.7	3.5	0.61	N = 42 * (5.7 / 3.5)^2^	111
TEA larger β	0.20	7.4	3.5	0.47	N = 31 * (7.4 / 3.5)^2^	139
*TEA updated larger* β*, smaller SD, smaller MIREDIF*	*0.20*	*5.7*	*3.0*	*0.53*	N = 31 * (5.7 / 3.0)^*2*^	*112*

The original and the updated TEA calculations and examples of different values of MERIDIF (minimally relevant difference), SD (standard deviation) and β (risk of type II error) are shown. As can be seen, the larger the MIREDIF you wish/expect to be able to detect or the smaller the estimated SD of the outcome measures or the larger the β, the smaller the sample size needed. In these calculations we used a two-sided α (risk of type I error) = 0.05 and we assumed SD_1_ = SD_2_. Effects sizes depend on MIREDIF and SD. Compared to the original calculation, the updated sample size calculation is balanced by allowing for a larger β, expecting a zsmaller SD and lowering the detectable MIREDIF. z = fractiles in normal distribution. ^#^N = 2 * (SD_1_ ^2^ + SD_2_ ^2^) * (z_α/2_ + z_β_)/MIREDIF^2^ ⇒ N = 4 * (z_α/2_ + z_β_)^2^ * (SD_1_/MIREDIF)^2^. The product 4 * (z_α/2_ + z_β_)^2^ is rounded up to whole figures in the equations.

Another challenge encouraging us to update our sample size estimation was the slow participant recruitment, as also demonstrated in other RCTs in child- and adolescent psychiatry [27]. Our original sample size (based on an estimated PANSS-P SD of 7.4, a MIREDIF of 3.5 and power of 90%) of n = 188 was expected to be recruited during a 2 year period from mid-2010. This expectation was based on available statistical data from the Danish Psychiatric Central Register concerning the incidence of first-episode psychosis in the involved geographical areas, whereof we expected to be able to randomise 50%. However, the recruitment turned out to be slower, primarily due to administrative difficulties in implementing the trial protocol in all centres. Adjustment of the sample size during the course of a clinical trial is a potential strategy to meet such challenges. We chose to update our sample size estimation by 1) adjusting the estimated SD of our primary outcome measure, change in PANSS-P, to a score of 5.7, based empirically on new relevant data from additional studies in the field that were relatively large and happened to report lower SDs, 2) changing the power from 90% to the more conventionally used 80%, and 3) lowering the MIREDIF from 3.5 to 3.0. As a result of these adjustments our updated sample size estimation still aims at detecting a difference between the two interventions on the primary outcome measure of a medium effect size of around 0.5, which is considered clinically relevant. At present, we have included all n = 112 patients during a recruitment period of 3½ years, and the last randomised patients are terminating the blinded treatment.

We believe that the updated sample size estimation is based on as accurate variables as possible, with the same conservative value for α, a value of SD, which is based on the most updated empirical body of evidence, and relevant estimates for the expected MIREDIF and effect size. We expect one agent being superior to the other by a score of 3 on the change in PANSS-P, which is in line with Arango et al. [20]. If the mean SD turns out to be higher than 5.7, we will use the statistical principles of interim analysis [114] to control for random errors. We will analyse the data by using the intention-to-treat principles and application of multiple imputations for missing data as outlined above.

### Additional outcomes

Apart from the primary and secondary outcomes stated above, we will explore additional perspectives of antipsychotic treatment in EOP. We find that the implication of psychosis and medication on social relations and everyday life needs to be better understood. One study among adolescents with mental disorders found that 62% experienced stigmatization (“being treated differently”) in relationships with peers and 46% by family members [115]. In TEA, the participants are followed up in naturalistic treatment settings after conclusion of the 3-month RCT. One year after randomisation we assess long-term health effects (same outcomes as at 12 weeks), and by means of qualitative interview techniques [116], we assess the patients’ perception of everyday life and of stigmatisation associated with psychosis and antipsychotic treatment.

Another issue that lacks attention in EOP are investigations of how the variation of genes coding for enzymes involved in drug metabolism or proteins involved in neurotransmission can influence drug response [117-119]. We will investigate how relevant gene variations are associated with serum concentration of antipsychotics (at week 4 and 12) and with benefits and harms of the antipsychotic treatment.

Finally, prognostic and background factors that could serve as moderators of outcome are assessed at baseline. These include premorbid functioning measured by the Premorbid Adjustment Scale (PAS) [120]; Duration of Untreated Psychosis (DUP) measured with the Instrument for the Assessment of Onset and Early Course of Schizophrenia (IRAOS) [121]), and intelligence measured with the Wechsler Intelligence Scale for Children (WISC-III/WISC-IV) [122,123], or Wechsler Intelligence Scale for adults (WAIS-III/WAIS-IV) [124,125].

Healthy controls, matched 2:1 to the randomised TEA patients on sex, age, and parental education, are also recruited in order to provide reference data for specific outcomes, including somatic assessments, cognitive function, and HRQoL measures.

### Strengths and limitations

The strengths of the TEA trial is the RCT design, the central handling of randomisation procedures, the comprehensive blinding, and the use of intention-to-treat analysis, all reducing the risks of bias [126,127]. The use of validated methods to assess benefits and harms increases the credibility of findings. The nationwide involvement of child and adolescent health centres aims at strengthening the generalizability of the TEA trial results.

A higher variation of the primary outcome measure than a priori expected may limit the statistical power of the trial. Furthermore, since sample size estimation is based on the primary outcome measure only, the effect sizes of differences between interventions on secondary measures are not a priori predicted, and we may not have enough power to conclude on all of our data. On the other hand, important adverse effects are likely to have larger effect sizes than the primary efficacy outcome, providing very likely sufficient power for these comparisons.

## Conclusion

The TEA trial addresses a significant gap in current research by conducting an independent randomised clinical trial with focus on the treatment of youth with severe mental illness. The TEA trial is expected to provide important and currently lacking information on the benefits and harms of two commonly prescribed, first-line antipsychotic compounds used to treat a vulnerable population. Results will improve our understanding of pharmacological effects of antipsychotics and will help guide clinical decision-making in the treatment of children and adolescents with psychosis.

## Abbreviations

AE: Adverse event; AIMS: Abnormal involuntary movement scale; BACS: Brief assessment of cognition in schizophrenia; BARS: Barnes akathisia rating scale; BRIEF: Behavioural rating inventory of executive functions; CGI-S/I/E: Clinical global impressions - severity/improvement/efficacy; DIPI: Dimensions of psychosis instrument; DUP: Duration of untreated psychosis; ECG: Electrocardiography; EMA: European medicines agency; EOP: Early onset psychosis; FDA: The food and drug administration; FGA: First-generation antipsychotic; GAPD: Global assessment of psychosocial disability; HRQoL: Health-related quality of life; IRAOS: The instrument for the assessment of onset and early course of Schizophrenia; K-SADS-PL: Schedule for affective disorders and schizophrenia for school-age children present and lifetime version; MIREDIF: Minimal relevant difference; PANSS: Positive and negative syndrome scale; PANSS-P: Positive and negative syndrome scale positive symptoms; PAS: Premorbid adjustment scale; RCT: Randomised clinical trial; SAS: Simpson angus scale; SCoRS-DK: Schizophrenia cognition rating scale, Danish version; SD: Standard deviation; SE: Standard error; SGA: Second-generation antipsychotics; TEA: Tolerability and Efficacy of Antipsychotics; UKU: Udvalget for kliniske undersøgelser side effect rating scale; VS: Versus; WAIS: Wechsler intelligence scale for adults; WISC: Wechsler intelligence scale for children.

## Competing interests

The authors Anne Katrine Pagsberg (AKP), Pia Jeppesen (PJE), Dea Gowers Klauber (DGK), Karsten Gjessing Jensen (KGJ), Ditte Rudå (DRU), Marie Stentebjerg-Olesen (MSO), Peter Jantzen (PJA), Simone Rasmussen (SRA), Eva Ann-Sofie Saldeen (EAS), Maj-Britt Glenn Lauritsen (MGL), Niels Bilenberg (NBI), Jesper Pedersen (JPE), Louise Nyvang (LNY), Sarah Madsen (SMA), Marlene B. Lauritsen (MBL), Ditte Lammers Vernal (DLV), Jacob Paludan (JPA), Thomas M. Werge (TMW), Kristian Winge (KWI), Klaus Juul (KJU), Christian Gluud (CG), Maria Skoog (MS), Jørn Wetterslev (JW), Jens Richardt M. Jepsen (JRJ), Birgitte Fagerlund (BFA) declare that they have no competing interests. Anders Fink-Jensen (AFJ) conducts an independent investigator- and university-initiated study supported by an unrestricted grant from Novo Nordisk. Christoph U. Correll (CC) has been a consultant and/or advisor to or has received honoraria from: Actelion, Alexza, Bristol-Myers Squibb, Cephalon, Eli Lilly, Genentech, Gerson Lehrman Group, IntraCellular Therapies, Janssen/J&J, Lundbeck, Medavante, Medscape, Merck, Otsuka, Pfizer, ProPhase, Roche, Sunovion, Supernus, Takeda, and Teva. He has received grant support from the American Academy of Child and Adolescent Psychiatry, BMS, Janssen/J&J, National Institute of Mental Health (NIMH), Novo Nordisk A/S, Otsuka, and the Thrasher Foundation, Anne Dorte Stenstrøm (ADS) has received honoraria from Otsuka. Per Hove Thomsen (PHT) has received speaker’s honoraria from Novartis, Shire, Medice and Janssen.

## Authors’ contributions

AKP, the study sponsor, has initiated the study and made important contributions to the study conception, design and protocol, and has led the manuscript drafting. BFA initiated and created the primary study protocol, made important contributions to the study conception and design, and has been involved in drafting and critically revising the manuscript. PJA, AFJ, CC, DGK, KGJ, DRU, MSO, CG, MS, JW, and JRJ have contributed significantly to the study conception, design and protocol, and have been involved in drafting and critically revising the manuscript. EAS, TMW, KWI, KJU, PJA, SR, MGL, NBI, ADS, JPE, LNY, SMA, MBL, DLV, PHT, and JPA have contributed to the study conception and in critically revising the manuscript. All authors approved the final version of the manuscript.

## Pre-publication history

The pre-publication history for this paper can be accessed here:

http://www.biomedcentral.com/1471-244X/14/199/prepub
